# Cadmium toxicity affects chlorophyll a and b content, antioxidant enzyme activities and mineral nutrient accumulation in strawberry

**DOI:** 10.1186/s40659-015-0001-3

**Published:** 2015-02-20

**Authors:** Ferhad Muradoglu, Muttalip Gundogdu, Sezai Ercisli, Tarik Encu, Fikri Balta, Hawa ZE Jaafar, Muhammad Zia-Ul-Haq

**Affiliations:** Department of Horticulture, Faculty of Agriculture and Natural Sciences, Abant Izzet Baysal University, Bolu, Turkey; Department of Horticulture, Faculty of Agriculture, Ataturk University, Erzurum, Turkey; Department of Horticulture, Faculty of Agriculture, Yuzuncu Yil University, Van, Turkey; Department of Horticulture, Faculty of Agriculture, Ordu University, Ordu, Turkey; Department of Crop Science, Faculty of Agriculture, University Putra Malaysia, 43400 Selangor, Malaysia; The Patent Office, Karachi, Pakistan

**Keywords:** Antioxidant enzymes, Cadmium, Chlorophyll, Heavy metal stress, Strawberry

## Abstract

**Background:**

Cadmium (Cd) is well known as one of the most toxic metals affecting the environment and can severely restrict plant growth and development. In this study, Cd toxicities were studied in strawberry cv. Camarosa using pot experiment. Chlorophyll and malondialdehyde (MDA) contents, catalase (CAT), superoxide dismutase (SOD), ascorbate peroxidase (APX) activities and mineral nutrient concentrations were investigated in both roots and leaves of strawberry plant after exposure Cd.

**Results:**

Cd content in both roots and leaves was increased with the application of increasing concentrations of Cd. We found higher Cd concentration in roots rather than in leaves. Chlorophyll a and b was decreased in leaves but MDA significantly increased under increased Cd concentration treatments in both roots and leaves. SOD and CAT activities was also increased with the increase Cd concentrations. K, Mn and Mg concentrations were found higher in leaves than roots under Cd stress. In general, increased Cd treatments increased K, Mg, Fe, Ca, Cu and Zn concentration in both roots and leaves. Excessive Cd treatments reduced chlorophyll contents, increased antioxidant enzyme activities and changes in plant nutrition concentrations in both roots and leaves.

**Conclusion:**

The results presented in this work suggested that Cd treatments have negative effect on chlorophyll content and nearly decreased 30% of plant growth in strawberry. Strawberry roots accumulated higher Cd than leaves. We found that MDA and antioxidant enzyme (CAT, SOD and APX) contents may have considered a good indicator in determining Cd tolerance in strawberry plant.

## Background

Cadmium is believed as one of the most important contaminant in the ecosphere. The main sources of Cd in environment are mining and smelting of Cd-containing ores, municipal wastes, pesticides, trace emissions, burning of fossil fuels and fertilizers [1,2]. In plants, the first organ to contact the toxic metal ions are roots, and therefore roots have greater contents of metal than aerial parts [2]. As compared to other metals like Zn, Cu or Mn, Cd is a non-essential heavy metal that is non-toxic at low concentrations, but it is toxic at higher concentrations [2]. It manifests its toxicity by inhibiting some growth, changing the plant nutrient contents and composition, and by antagonizing the effects on essential elements and several enzymes activities [3,4]. It induces complex changes in plants at genetic, physiological and biochemical levels, leading to phytotoxicity, whose main indications are leaf rolls, chlorosis and reduction of root and stem growth [5,6], limiting transport of metals [7], respiratory and photosynthetic activities, enzyme activities, hormone balance and membrane functions [8], induction of lipid peroxidation and chlorophyll breakdown in plants [9] and generation of oxidative stress [2,10]. Among all side effects induced by Cd, lipid peroxidation is the most harmful as it can lead to bio membrane deterioration. The main indicator of oxidative stress in plants is MDA, which is the decomposition product of polyunsaturated fatty acids of bio membrane [11]. Plants manage the oxidative stress by antioxidant enzymes like CAT, SOD, GPX, APX, GR, and non-enzymatic constituents such as ascorbate and glutathione [12-14]. Among enzymes, SOD is the first line of defense as it converts superoxide radical to hydrogen peroxide (H_2_O_2_), which is later reduced to water and oxygen either by APX in ascorbate-glutathione cycle or by GPX and CAT in cytoplasm and other cellular compartments [12]. It is well known that the response of plants to Cd-induced depend on several factors such as genotype, root system, growing condition, agronomic practices employed, climatological and geological conditions of soil, and growing season as well as maturity of plants. Root uptake, root-to-shoot translocation and partitioning of Cd between plant organs can vary in both plant species and cultivar belongs to single specie [15].

Strawberry (*Fragaria x ananassa* Duch) has been widely grown worldwide because of adapting to various climate and soil condition. Camarosa cultivar dominates strawberry production in Turkey due to its bigger fruits, high fruit quality and excellent transportation capacity [16,17]. Threats of environmental pollution with heavy metals render stress a general concern for the agricultural crops. Strawberry plants exposed to Cd toxicity may experience severe cellular injury that may lead to cell death within a short period. Cd is easily taken up by strawberry plants and accumulated in organs [18]. Previous studies commonly concerned with the influences of Cd on the upper part of plants. Little is known of Cd toxicity to the root system in strawberry plant. This study was an attempt to understand the effect of Cd treatments on plant growth, antioxidant enzyme activities and mineral nutrients accumulations in both roots and leaves of Camarosa strawberry cultivar.

## Results and discussion

### Effect of Cd on chlorophyll and Cd accumulation in strawberry

**Figure 1:**
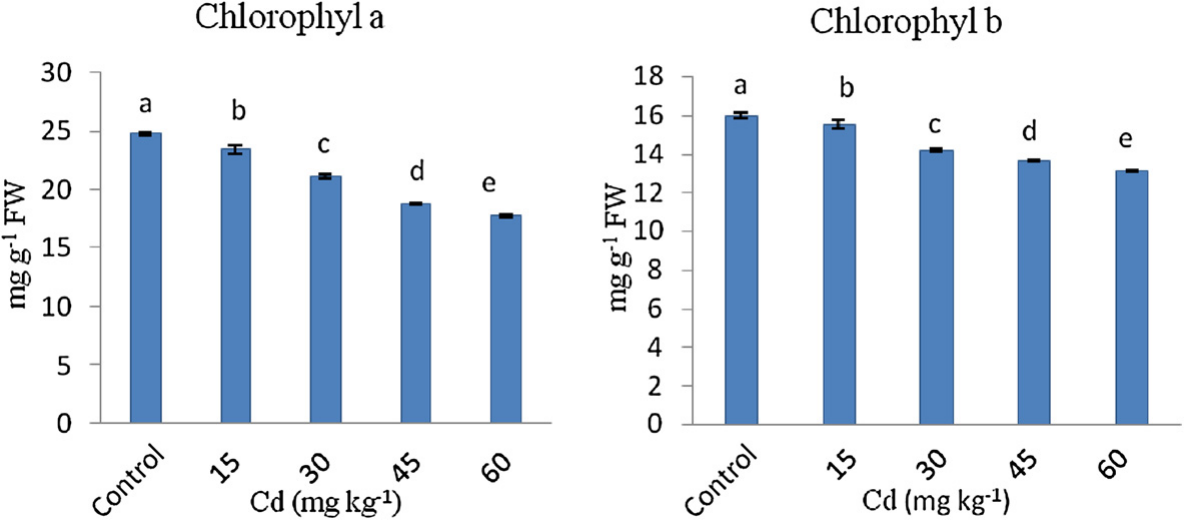
**Changes of chlorophyll a and b contents exposed to different Cd applications in leaves of strawberry plants.** Same letters are not significantly different according to Duncan test (p≤0.05).

As shown in Figure 1. The chlorophyll content in strawberry plant organs decreased under Cd treatment. There was regularly a reduction attributable to Cd application both chlorophyll a and chlorophyll b in Camarosa (strawberry) cultivar. Chlorophyll a content was found higher than chlorophyll b content. There was nearly 5, 15, 25, and 30% decrease in chlorophyll a and 3, 11, 15 and 18% decrease in chlorophyll b when Cd applications were increased from 0 to 60 mg kg^−1^ respectively. According to Qian et al. [19], cadmium-induced declining effect on chlorophyll and carotenoid contents which could be explained on the basis of inhibitory effect of Cd on enzymes involved in pigment biosynthesis. Furthermore, chlorophyll a and chlorophyll b contents showed significant decline at the applications of Cd and the results were in consist with earlier report where Cd inhibited the biosynthesis of chlorophyll and generated a kind of senescence [19,20]. Our results are in agreement with finding by Yang et al. [21] who reported that leaves of *Potamogeton crispus* under Cd stress showed decreased 35,8% chlorophyll a and 26.7% chlorophyll b and chlorophyll a content was found higher than chlorophyll b content. Several report have shown that under Cd stress decrease chlorophyll content in leaf garden grass [22] and almond seedling [23]. Therefore, chlorophyll pigments seem to be one of the main reasons of heavy-metal injury in plants.

**Figure 2:**
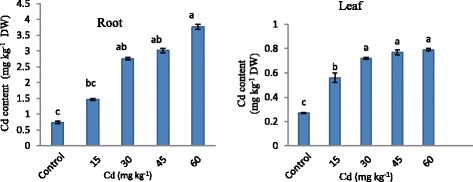
**Cadmium accumulation in strawberry plant exposed to different Cd applications.** Same letters are not significantly different according to Duncan test (p ≤ 0.05).

Statistically significant differences among Cd applications for accumulation of Cd in root and leaf of strawberry plants were observed (Figure 2). Increasing Cd concentrations were ensuring significant increase Cd accumulation in both root and leaf. The average Cd concentration in root was approximately four times higher than in leaf. The Cd concentration ranged from 0.74 to 3.77 mg kg^−1^ in root and from 0.27 to 0.79 mg kg^−1^ in leaf. Increasing Cd concentrations were increased accumulation of Cd approximately 1.98, 3.72, 4.08 and 5.09 times in root and 2.07, 2.26, 2.85 and 2.92 times in leaf as compared with control respectively. Cd uptake and accumulation in plant differences greatly among species and also among different organs and tissues. Cd is usually accumulated in the roots, because this is the first organ exposed to heavy metal and it is also translocated into the shoots. Our results showed that the accumulation of Cd in root was higher than in leaf of strawberry (Figure 2). Similarly, Gill et al. [22] reported that Cd accumulation in root and leaves increased with the increasing Cd concentration in soil and Cd content in root was found higher than leaves in *Lepidium sativum*. Nada et al. [23] observed similar situation in almond seedling.

### Effects of Cd on MDA content

**Figure 3:**
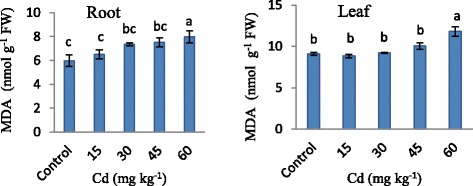
**Changes in malondialdehyde content in strawberry plant exposed to different Cd applications.** Same letters are not significantly different according to Duncan test (p ≤ 0.05).

The increased contents of lipid peroxides are indication of more production of toxic oxygen species than normal. Strawberry plant showed significant increase in MDA production when treated with Cd applications. Leaf had higher MDA content than root. In root and leaf of strawberry plant, MDA production was increased nearly 30% in root and 33%in leaf compare with control after expose to 60 mg kg^−1^ Cd application (Figure 3). When plants grow in stressed environments, free-radicals generated in excess, accumulate in the cells. It leads to lipid peroxidation of biomembranes, and its end product is MDA. Therefore, the MDA-concentration is an indicator of physiological stresses and the aging process [8]. Our results showed increase in MDA content in both root and leaf depend on Cd concentrations. Nada et al. [23] observed an increase MDA content in both root and leaf of almond seedlings that exposed to Cd treatment. This result is in agreement with our study.

### Effect of Cd on antioxidant enzyme activity

**Figure 4:**
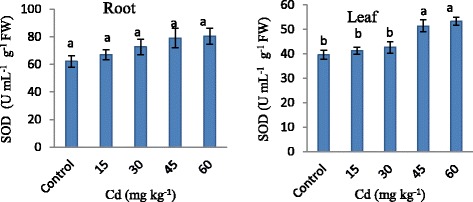
**SOD activity in strawberry plant exposed to different Cd applications.** Same letters are not significantly different according to Duncan test (p ≤ 0.05).

The changes in SOD activity were determined in both root and leaf of strawberry with increase in level of Cd concentrations when compared with control (Figure 4). With increase in Cd concentration in strawberry plant, a steadily increase in SOD was determined in both root and leaf. In every Cd concentration increase, SOD activity was higher than control. In root, Cd concentrations caused an increase in SOD activity by 8, 17, 27 and 29% respectively and in leaf 4, 7, 29 and 34% as compared with control.

**Figure 5:**
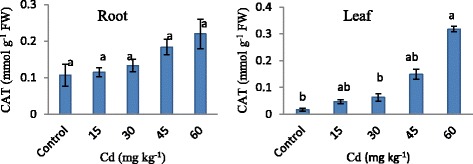
**CAT activity in strawberry plant exposed to different Cd applications.** Same letters are not significantly different according to Duncan test (p ≤ 0.05).

As shown in Figure 5, significant increases were observed in CAT activity. Increasing Cd concentrations provide to regularly increase CAT activity in root but Cd concentrations provide a severe increasing in leaf. When the increasing CAT activity was compared with to Control, increasing Cd concentrations caused an increase in CAT activity by 1.0, 1.2, 1.7 and 2.0 times in root respectively but this increase was followed very sharp by 3, 4, 9 and 19 times in leaves.

**Figure 6:**
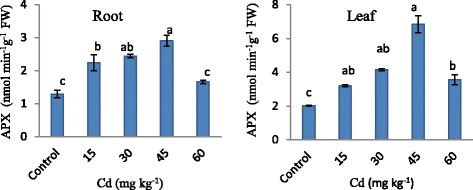
**APX activity in strawberry plant exposed to different Cd applications.** Same letters are not significantly different according to Duncan test (p ≤ 0.05).

APX results are shown in Figure 6. Increasing in APX activity belongs to Cd concentrations was found statistically significant. APX activity was monitored regularly increased by 124% in root and 237% in leaf up to 45 mg kg^−1^ Cd concentration as compared control. On the other hand, a decline in APX activity was monitored after 45 mg kg^−1^ Cd concentration that this decline was higher than the control by 28% in root and 74% in leaf. APX activity in leaf was higher than in root.

The abiotic stresses like heavy metals lead to molecular damage to plant cells by generating reactive oxygen species (ROS) [10]. Although Cd does not generate ROS directly, it generates oxidative stress by interrupting the antioxidant defense system [24]. Produced these ROS mainly include GPX, APX and CAT. These antioxidant enzymes balance the ROS production and destruction. Cd also inhibits Calvin cycle enzymes and hence accumulated reduced coenzymes will not be able to accept electrons from PSI. In our experiment the activities of catalase, ascorbate peroxidase and superoxide dismutase were measured. Our results showed that 15, 30, 45 and 60 mg kg^−1^ Cd concentrations led to a significant increase in the antioxidant enzyme activity (SOD; CAT; APX) in both root and leaf of strawberry (Figures 4, 5 and 6). Our results are in agreement with previous studies that have observed findings of Gill et al. [22] who reported that activities of SOD, CAT and APX were found increased in the leaves of garden gress plant with increased dose of Cd treatment.

### Effect of Cd on mineral concentration of strawberry

Increasing of Cd concentrations affected content of mineral elements in Camarosa cultivar. In leaves, K, Mg and Mn content was found higher than in root, but Fe, Cu and Zn content was found higher in root with increasing Cd concentrations (Table 1). In leaves, contents of essential elements (Ca, Mg, Fe, Mn, Cu and Zn) were found statistically significant according to Cd concentrations except K while Mg, Fe, Mn and Zn was found statistically significant in root based on Cd concentrations. Initially K, Ca, Mg Fe and Mn contents were tending to increase when compare with control then a slight decrease was observed in both root and leaf at 60 mg kg^−1^ Cd concentration. With increasing Cd concentration Zn, Cu and Mn content was observed decrease in both root and leaf except Cu in root. Nada et al. [23] found that in leaf and root of almond, Cd addition reduced the concentration of macronutrients such as Ca, Mg and K in leaves and in root. Liu et al. [4] report that the interactions of Cd and Fe, Cu and Zn are synergetic in uptake and translocation from root to shoot by rice plants. Yang et al. [21] also reported that a decrease macronutrient (K and P) contents in *Potamogeten crispus*.

**Table 1:** **Effect of Cd applications on macro-micro nutrient elements concentrations of root and leaf of strawberries Camarosa cultivars (mg kg** ^**-1**^ **DW)**

**Cd applications**		**Control**	**15**	**30**	**45**	**60**
	Root	3291^a^	3608^a^	4698^a^	4679^a^	3784^a^
K	Leaf	18655^a^	15325^a^	16339^a^	18846^a^	14921^a^
	Root/Leaf	0.17	0.23	0.28	0.24	0.25
	Root	17144^a^	17972^a^	18769^a^	16295^a^	15695^a^
Ca	Leaf	11842^c^	15489^c^	21105^ab^	22714^a^	16302^bc^
	Root/Leaf	1.45	1.16	0.89	0.72	0.96
	Root	4038^a^	4134^a^	4215^a^	3257^b^	2930^b^
Mg	Leaf	4779^b^	6037^ab^	6759^ab^	7704^a^	6646^ab^
	Root/Leaf	0.85	0.68	0.62	0.42	0.44
	Root	980^b^	1333^ab^	1653^a^	1439^ab^	1326^ab^
Fe	Leaf	203^b^	191^b^	251^a^	190^b^	184^b^
	Root/Leaf	4.82	6.96	6.59	7.58	7.19
	Root	38^c^	38^c^	60^b^	81^a^	31^c^
Mn	Leaf	105^d^	148^c^	202^b^	230^ab^	261^a^
	Root/Leaf	0.37	0.26	0.30	0.35	0.12
	Root	15.49^a^	16.38^a^	17.10^a^	18.04^a^	20.65^a^
Cu	Leaf	10.99^ab^	8.63^bc^	8.60^bc^	13.39^a^	6.45^c^
	Root/Leaf	1.41	1.9	1.99	1.35	3.2
	Root	206^a^	52.87^c^	72.94^b^	40.30^d^	35.63^d^
Zn	Leaf	22^a^	17.69^b^	17.49^b^	19.32^ab^	18.90^ab^
	Root/Leaf	9.34	2.98	4.17	2.08	1.88

Same letters in the same line are not significantly different according to Duncan test (p≤0.05).

## Conclusions

The results suggested that increasing Cd concentrations had negative effect on chlorophyll content and nearly decrease 30% in leaves. The roots accumulate about higher 70% Cd than leaves of strawberry. Results indicated that MDA and antioxidant enzymes (SOD, CAT and APX) content are considered to be indicator in determining Cd tolerance in plant. Strawberry plants affected with increased Cd concentrations. Lipid peroxidation content and antioxidant enzyme activities increased with Cd concentrations.

## Methods

### Plant materials and pot experiment

The experiment was carried out in the greenhouse of Yuzuncu Yil University during growing period (from middle May to end of July). The experiment was conducted by using frigo plants of Strawberry (*Fragaria x ananassa* cv. Camarosa) in pot experiment. Four frigo plants were planted into every pot (72x20x17cm) that was filled with peat (4 kg) [25]. Initial stages of grown, plants were fed by adding nutrient solution to the pots. The nutrition solutions contained N 200, Mg 49, K 208, P 37, Ca 167, Mn 1.16, Fe 1.53, Zn 0.09, B 0.46, Cu 0.03 and Mo 0.02 mg/l. Flower buds were cut of early stage of plant’s growth. After the plants had four or five leaves about 4 weeks, cadmium applications were started. Cadmium was added to pots at concentration of 0, 15, 30, 45 and 60 mg kg^−1^ in the form of CdSO_4_*8 H_2_O four equal times with watering during growth period. In harvest, 12 plants were harvested to every application, plants were sectioned into roots and leaves and this section was stored at −80°C until antioxidant analyze. Also for macro–micro analysis, fresh root and leafs dried in an oven (80°C) and dried parts were ground and stored until analyze.

### Chlorophyll determination

Chlorophyll a and chlorophyll b, 0.5 g fresh leaves were extracted in 80% acetone and were determined spectrophotometrically by Lichtentaler formula [26].

### Lipid peroxidation content

MDA content, a product of lipid peroxidation, was used to gauge the level of lipid peroxidation [27]. A leaf sample (0.5 g) was homogenized in trichloro acetic acid, TCA (10 ml; 0.1%). The homogenate was centrifuged (15 000 g; 5 min) and supernatant was collected. To aliquot (1.0 ml) of the supernatant, 4 ml of 0.5% thiobarbituric acid (TBA) in TCA (20%) was added. The mixture was heated at 95°C for half an hour and then quickly cooled in an ice bath. After centrifugation (10 000 g; 10 min), the absorbance of the supernatant was recorded at 532 nm. The value for non-specific absorption at 600 nm was subtracted. The MDA content was calculated by its extinction coefficient of 155 mM^−1^ cm^−1^ and expressed as nmol MDA per gram fresh weight.

### Preparation of extracts and determination of antioxidant enzymes

For the analysis of antioxidant enzyme, 1 g fresh tissue from fourth leaves and the roots was homojenized in 5 ml cold 0.1 M 0.1 M Na-phospat, 0.5 mM Na-EDTA and 1 mM ascorbic acid (pH: 7.5). Samples were centrifuged at 18 000 g for 30 min at a temperature 4°C. Then Catalase activity immediately was determined and the supernatant was stored at −20°C until determined for SOD.

CAT activity was determined using the modified Aebi [28] method, by measurement of the decrease in absorbance at 240 nm for 2 min, in a solution containing H_2_O_2_ (10 mM) in phosphate buffer (pH 7.0; 50 mM). Enzyme activity was defined as the consumption of 1 μmol H_2_O_2_ per min and mL using a molar absorptivity of 39.4 mM^−1^ cm^−1^.

SOD activity was measured by monitoring the inhibition of nitroblue tetrazolioum (NBT) reduction at 560 nm as reported by Giannopolitis and Ries [29]. The reaction mixture contained phosphate buffer (pH 7; 50 mM), Na-EDTA (0.1 mM), riboflavin (75 μM), methionine (13 mM) and enzyme extract (0.1-0.2 ml). Reaction was carried out in test tubes at 25°C under fluorescent lamp (40 W) with irradiance of 75 μmol m^−2^ s^−1^. The reaction was allowed to run for 10 min and stopped by switching the light off. Blanks and controls were run similarly but without irradiation and enzyme, respectively. Under the experimental condition, the initial rate of reaction, as measured by the difference in increase of absorbance at 560 nm in the presence and absence of extract, was proportional to the amount of enzyme.

APX activity was assayed according to the method of Nakano and Asada [30] by recording the decrease in ascorbate content at 290 nm, as ascorbate was oxidized. The reaction mixture contained potassium phosphate buffer (pH 7.0; 50 mM), ascorbic acid (5 mM), EDTA (0.1 mM), H_2_O_2_ (0.1 m M) and diluted enzyme (0.1 ml) in a total volume of 3.0 ml. The reaction was started with the addition of H_2_O_2_ and absorbance was recorded at 290 nm spectrophotometrically for 1 min.

### Macronutrient and micronutrient determination

İn dried leaves and roots, Cd contents and others nutrient element concentrations were analyzed by an atomic absorption spectrophotometer (Varian Techtron Model AAS 1000, Varian Associates, Palo Alto, CA). The samples, which were digested in an acid solution (HCL 3%) were passed through the AAS system using different lamps, and calibrated with related minerals in different concentrations for different micronutrients.

### Statistical analysis

The experiment was designed as a complete random block design and all measurements were replicated four times. The statistical analysis of the data obtained was performed using the software SPSS 22.0. The results were subjected to one-way ANOVA using the Duncan test to check for significant differences between means (p < 0.05). Error bars in graphs represent ± standard error.
